# Self-Nanoemulsifying Drug Delivery System Loaded with *Psiadia punctulata* Major Metabolites for Hypertensive Emergencies: Effect on Hemodynamics and Cardiac Conductance

**DOI:** 10.3389/fphar.2021.681070

**Published:** 2021-06-10

**Authors:** Hossam M. Abdallah, Hany M. El-Bassossy, Ali M. El-Halawany, Tarek A. Ahmed, Gamal A. Mohamed, Azizah M. Malebari, Noura A. Hassan

**Affiliations:** ^1^ Department of Natural Products and Alternative Medicine, Faculty of Pharmacy, King Abdulaziz University, Jeddah, Saudi Arabia; ^2^ Department of Pharmacognosy, Faculty of Pharmacy, Cairo University, Cairo, Egypt; ^3^ Department of Pharmacology, Faculty of Pharmacy, Zagazig University, Zagazig, Egypt; ^4^ Department of Pharmaceutics, Faculty of Pharmacy, King Abdulaziz University, Jeddah, Saudi Arabia; ^5^ Department of Pharmaceutics and Industrial Pharmacy, Faculty of Pharmacy, Al-Azhar University, Cairo, Egypt; ^6^ Department of Pharmacognosy, Faculty of Pharmacy, Al-Azhar University, Assiut Branch, Assiut, Egypt; ^7^ Department of Pharmaceutical Chemistry, College of Pharmacy, King Abdulaziz University, Jeddah, Saudi Arabia

**Keywords:** methoxy flavonoids, umuhengerin, reflex tachycardia, vasodilators, SNEDDS

## Abstract

Vasodilators are an important class of antihypertensive agents. However, they have limited clinical use due to the reflex tachycardia associated with their use which masks most of its antihypertensive effect and raises cardiac risk. Chemical investigation of *Psiadia punctulata* afforded five major methoxylated flavonoids (**1–5**) three of which (**1**, **4**, and **5**) showed vasodilator activity. Linoleic acid-based self-nanoemulsifying drug delivery system (SNEDDS) was utilized to develop intravenous (IV) formulations that contain compounds **1**, **4**, or **5**. The antihypertensive effect of the prepared SNEDDS formulations, loaded with each of the vasodilator compounds, was tested in the angiotensin-induced rat model of hypertension. Rats were subjected to real-time recording of blood hemodynamics and surface Electrocardiogram (ECG) while the pharmaceutical formulations were individually slowly injected in cumulative doses. Among the tested formulations, only that contains umuhengerin (**1**) and 5,3′-dihydroxy-6,7,4′,5′-tetramethoxyflavone (**5**) showed potent antihypertensive effects. Low IV doses, from the prepared SNEDDS, containing either compound **1** or **5** showed a marked reduction in the elevated systolic blood pressure by 10 mmHg at 12 μg/kg and by more than 20 mmHg at 36 μg/kg. The developed SNEDDS formulation containing either compound **1** or **5** significantly reduced the elevated diastolic, pulse pressure, dicrotic notch pressure, and the systolic–dicrotic notch pressure difference. Moreover, both formulations decreased the ejection duration and increased the non-ejection duration while they did not affect the time to peak. Both formulations did not affect the AV conduction as appear from the lack of effect on *p* duration and PR intervals. Similarly, they did not affect the ventricular repolarization as no effect on QTc or JT interval. Both formulations decreased the R wave amplitude but increased the T wave amplitude. In conclusion, the careful selection of linoleic acid for the development of SNEDDS formulation rescues the vasodilating effect of *P. punctulata* compounds from being masked by the reflex tachycardia that is commonly associated with the decrease in peripheral resistance by most vasodilators. The prepared SNEDDS formulation could be suggested as an effective medication in the treatment of hypertensive emergencies, after clinical evaluation.

## Introduction

Hypertension is one of the most common chronic medical problems affecting more than one billion people all over the world (Chockalingam et al., 2006). The “silent” nature of hypertension can lead a majority of hypertensive patients to the emergency room with a hypertensive emergency, an important clinical entity, which if not recognized and acted upon promptly, can result in life-threatening adverse outcomes (Mallidi et al., 2015). Vasodilators form the mainstay of treatment in hypertensive emergencies (Mallidi et al., 2015) and a valuable agent in the treatment of systemic hypertension (Hariri and Patel, 2021). However, they have a limited clinical role due to the reflex tachycardia associated with their use, as a consequence of the medication-induced baroreflex response compensating for the sudden medication decrease in vascular resistance (Kester et al., 2012; Rascher and Paech, 2020), furthermore, reflex tachycardia might propagate aortic dissection (Estrera et al., 2006).

Angiotensin II, the major biologically active component of the renin-angiotensin system, contributes to the regulation of vascular tone, salt and water balance, and blood pressure (Kurdi and Booz, 2011; Cuthbert et al., 2020). Angiotensin II is well known for its strong vasoconstricting effect that leads to an increase in blood pressure (Rascher and Paech, 2020). Infusion experiments using angiotensin II establish a qualitative and quantitative correlation between elevated concentrations of plasma angiotensin II and elevated arterial pressure (White et al., 1989).

In recent years, there has been a growing interest in the use of bioactive compounds from plant sources to treat hypertension, owing to their efficacy, safety, relative availability, and low cost (Verma et al., 2020). Different phytoconstituents, including flavonoids, alkaloids, and terpenoids have been identified as vasodilators from plants (Micucci et al., 2020).

*Psiadia punctulata* (DC.) Vatke (Asteraceae) is a small shrub mostly found in some east African countries including Eritrea, Saudi Arabia, and North East India (Kidane et al., 2018). Methanol extract of the whole plant is reported to show a blood pressure-lowering effect, phrenic neuromuscular nerve blocking effect and relaxant properties on the smooth muscle of mice trachea (Achola et al., 1998). In our previous work, we have isolated five methoxylated flavonoids from *P. punctulata*, out of which three compounds namely; umuhengerin (**1**), luteolin-3′,4` -dimethyl ether (**4**), and 5,3′-dihydroxy-6,7,4′,5′-tetramethoxyflavone (**5**) have produced significant vasodilation which is mediated through endothelial nitric oxide pathway (Abdallah et al., 2020). However, the utilization of the previously isolated vasodilators in treating hypertension invivo has not been examined yet.

Linoleic acid is an essential *n*-6 polyunsaturated fatty acid required for normal growth and development at 1–2% of daily energy (Taha, 2020). It has been used in the development of many drug delivery systems such as liposomes, in the treatment of melasma (Irby et al., 2017); nanoemulsion, to enhance oral bioavailability of simvastatin (Singh et al., 2016); and the development of self-nanoemulsifying drug delivery system (SNEDDS), to improve quercetin hepatoprotective activity (Van Staden et al., 2020). Previous reports suggest that linolenic acid may protect against coronary artery disease and related death (Marangoni et al., 2020). The linoleic acid protection was attributed to its effect on heart rate suggesting possible antiarrhythmic activity (Christensen et al., 2005).

The present study aimed to evaluate the antihypertensive activity for the isolated methoxylated flavonoids from *P. punctulata* after administration of an IV formulation based on SNEDDS containing linoleic acid to unmask the effect of reflex tachycardia.

## Materials and Methods

### Material

Polyethylene glycol (PEG) 400, oleic acid, and Tween® 80 were purchased from Sigma–Aldrich (St. Louis, MO). Linoleic acid was obtained from Acros organics (Fair Lawn, New Jersey). Angiotensin II and dimethyl sulfoxide (DMSO) from (Sigma-Aldrich, Munich, Germany) were used in the biological study.

### Plant Material and Isolation of Active Compounds

The methods of collection, extraction, and isolation of the methoxy flavonoids from the flowering aerial parts of *P. punctulata* were mentioned in detail in our previous publication (Abdallah et al., 2019).

### Development of IV Formulation

#### Preparation of Ternary Phase Mixtures

In this study, self-nanoemulsifying drug delivery system (SNEDDS) was utilized to develop the IV formulation. PEG 400 was selected as a cosurfactant, tween 80 (polysorbate 80) was selected as a surfactant. Three oils namely, olive oil, linoleic acid, and oleic acid were screened to select the oil that produces SNEDDS with the lowest globule size. The selection of the surfactant and cosurfactant was based on our previously published work (Ahmed et al., 2014; El-Say et al., 2015; Ahmed et al., 2018). The selection of the oils was based on their biocompatibility that renders the formulation suitable for IV administration. Seven different formulations containing different quantities of oil, surfactant, and cosurfactant were proposed and their composition is illustrated in Table 1. Briefly, 1 g of SNEDDS was prepared by accurately weighing the calculated amount of oil, surfactant, and cosurfactant in an Eppendorf tube. Each mixture was vortex for 30 s until a homogenous dispersion was obtained. The total weight of the oil, surfactant and cosurfactant in any SNEDDS mixture was always added to 100%.

**TABLE 1:** Composition of the prepared self-nanoemulsifying drug delivery system (SNEDDS) and the obtained results for the size and polydispersity index.

Run	Oil %	PEG 400%	Tween 80%	Size (nm)			PDI		
				Olive oil SNEDDS	Linoleic acid SNEDDS	Oleic acid SNEDDS	Olive oil SNEDDS	Linoleic acid SNEDDS	Oleic acid SNEDDS
1	10	80	10	422 ± 15.62	233.33 ± 4.04	367 ± 14.73	0.897 ± 0.059	0.411 ± 0.032	0.889 ± 0.08
2	20	70	10	662.33 ± 59.80	446.33 ± 10.02	1,095.33 ± 130.27	0.950 ± 0.036	0.909 ± 0.039	0.857 ± 0.13
3	30	60	10	1,170.33 ± 142.46	793.67 ± 30.66	1,288 ± 70.92	0.940 ± 0.043	0.507 ± 0.015	0.940 ± 0.04
4	40	50	10	1716.33 ± 225.75	865.33 ± 48.88	1,690.67 ± 111.21	0.916 ± 0.081	0.972 ± 0.038	0.916 ± 0.08
5	50	40	10	5,782.67 ± 431.37	1,104 ± 59.19	2,114.67 ± 203.85	0.561 ± 0.107	0.929 ± 0.097	0.995 ± 0.04
6	20	60	20	775 ± 81.05	333.33 ± 8.73	457 ± 39.94	0.994 ± 0.004	0.549 ± 0.094	0.834 ± 0.03
7	30	50	20	2064.33 ± 290.59	439.67 ± 22.47	631.33 ± 57.38	0.830 ± 0.146	0.820 ± 0.035	0.975 ± 0.03

#### Characterization of the Prepared SNEDDS

Known weight (1 g) of the prepared SNEDDS formulation was added to 20 ml of distilled water on a magnetic stirrer. Stirring was continued until the formation of a homogenous dispersion (nano-emulsion). The globule size and polydispersity index (PDI) of the obtained emulsions were determined using Malvern Zetasizer Nano ZSP, Malvern Panalytical Ltd. (Malvern, United Kingdom). Dynamic light scattering with non-invasive backscatter optics was the technique used to measure the size. An average of three readings was recorded.

#### Preparation of the Medicated IV Formulation

Known weight (2.1 mg) of the freeze-dried isolated compounds was separately added to 1 g of the selected SNEDDS formulation which contains 10% linoleic acid, 80% PEG 400, and 10% tween 80. The mixture was vortex until the complete dissolving of the compound in the SNEDDS formulation. The medicated SNEDDS (1 g) was added to 20 ml double distilled water on a magnetic stirrer. Stirring was continued until the formation of a homogenous mixture that was sterile filtered using Ministar® single-use 0.45 mm, non-pyrogenic syringe filter of Sterile-ED, Sartorius Stedim Biotech GmbH (Goettingen, Germany) to prepare medicated IV formulations. Plain (non-medicated) IV formulation was also prepared for comparative study.

### Biological Study

#### Animals

Six-week-old male Wistar rats with a weight of (250–275 g) were obtained from Zagazig University. They were kept in clear cages made of polypropylene and with good ventilation (3–4 rats in each cage), under constant environmental conditions of 22 ± 2°C temperature, 50–60% relative humidity, and 12-h day and night cycle. Unlimited rodent pellet food and purified water were provided to the rats. The experimental design and animal handling procedures were as indicated by the guidelines of the Ethical Committee for Animal Handling at Zagazig University (approval number ZU-IACUC/3/F/33/2021).

#### Blood Pressure Recording

The blood pressure was recorded invasively in real-time following the procedure outlined in our previous publications (Azhar and El-Bassossy, 2014; El-Bassossy and Watson, 2015; El-Bassossy et al., 2017b; El-Bassossy et al., 2017c). The rats were subjected to anesthesia with a single intraperitoneal injection of 100 mg/kg ketamine and 10 mg/kg xylazine. Animals’ body temperature was held at 37°C through a rectal probe and automated heating pads. A micro-tip pressure-volume catheter (PV catheter, SPR-901, Millar Instruments, Houston, TX, United States of America) was inserted via a small opening into the right carotid artery. This instrument can continuously monitor arterial pressure. The micro-tip catheter was linked via a Power Lab Data Interface to a computer running the Lab Chart professional software (v8.0, AD Instruments, Bella Vista, Australia) incorporating a blood pressure (BP) module. Following a stabilization time of 5 min, readings were continuously recorded. The BP module was employed to real-time monitor all hemodynamic parameters including; systolic BP, diastolic BP, heart rate, pulse pressure, dicrotic notch pressure, ejection duration, non-ejection duration and time to peak.

#### Electrocardiogram (ECG) Recording

A Powerlab® system (AD Instruments, Bella Vista, Australia) linked to a computer running the LabChart professional software with the ECG module was employed to record the standard surface ECG according to the methodology outlined in a previous report by our group (El-Bassossy et al., 2016; El-Bassossy et al., 2017a; El-Bassossy et al., 2018). The ECG module quantitatively assesses the various elements of the ECG including; *p* duration, PR interval, QTc interval, JT interval, R amplitude and T amplitude.

#### Acute Induction of Hypertension

After 10 min (stabilization period) of basal recording of invasive blood pressure and ECG, the standard dose of angiotensin II (120 ng/min/kg) that were commonly used in osmotic mini-pumps (Gonzalez et al., 2014; El-Bassossy et al., 2018) was slowly infused through the femoral vein using a syringe pump and continued throughout the experiment duration.

#### Animals Treatment

Animals were divided into 4 groups each of 6 rats: saline group, vehicle (the plain IV formulation), IV formulation containing compound **1** group, and IV formulation containing compound **5** group. After 10 min (stabilization period) of starting angiotensin infusion; the saline and the plain and the medicated IV formulations containing either compound **1** or **5** were injected into the femoral vein in doses of 12, 24, 36 μg/kg every 10 min each in 0.1 ml injection volume. Invasive blood hemodynamics and ECG were continuously recorded throughout the experiment. Saline (0.3 ml) was injected in time control experiments.

#### Effects on Serum Sodium and Potassium Levels

At the end of the experiment, blood samples were obtained from the femoral vein. Levels of sodium and potassium in blood were analyzed by colorimetric methods using a spectrophotometer using the commercially available kits. The method is based on the reaction of sodium or potassium with a selective chromogen producing a chromophore whose absorbance varies directly as the concentration of sodium or potassium in the test specimen.

#### Statistical Analysis

Values of the present study presented in form of mean ± SEM. Statistical analysis was carried out using the Prism 5 computer program (Graph Pad, United States). Statistical comparison of blood hemodynamics and electrocardiogram parameters was done using repeated measures Two-way analysis of variance (ANOVA) followed by Bonferroni’s post-hoc test of baseline-corrected data, while the statistical comparison of electrolyte levels was done by using one way ANOVA, followed by Newman–Keuls’ post hoc test. *p* < 0.05 was considered significant.

## Results and Discussion

To the best of our knowledge, the current study is the first to report a potent antihypertensive activity for a linoleic acid-based SNEDDS IV formulation containing methoxy flavonoid (umuhengerin or 5,3′-dihydroxy- 6,7,4′,5′-tetramethoxyflavone), isolated from *P. punctulata*. The formulation caused a significant antihypertensive effect through vasodilatation and a decrease in peripheral resistance without reflex tachycardia. This behavior could be detected through the ability of the developed formulation to reduce the elevated diastolic, pulse pressure, dicrotic notch pressure, and the systolic-dicrotic notch pressure difference. The developed IV formulation could be considered as an effective medication in the treatment of hypertensive emergencies, after clinical evaluation.

### Characterization of Isolated Compounds

**FIGURE 1:**
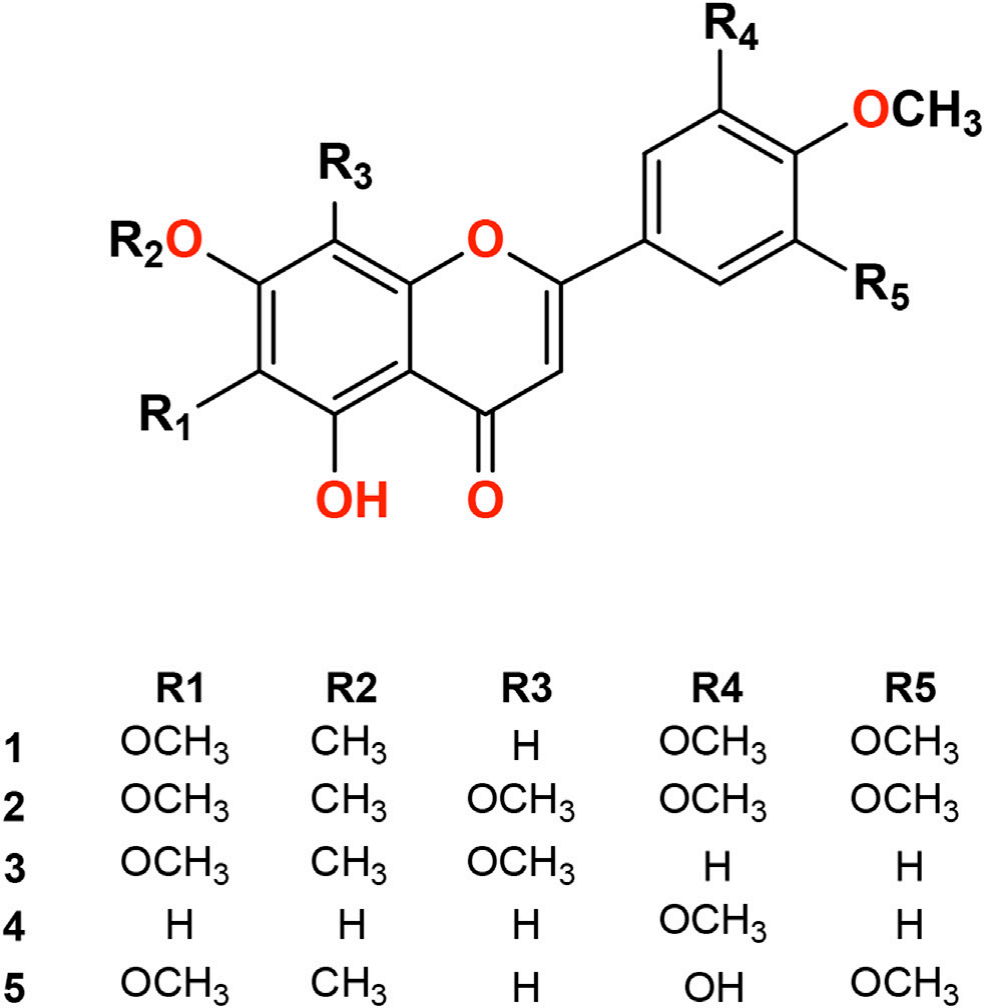
Isolated compounds (**1-5**) from *Psiadia punctulata*.

Isolated compounds from *P. punctulata* were identified based on their NMR data and comparison with previously published data. The compounds (Figure 1) were identified as umuhengerin (**1**), gardenin A (**2**), gardenin B (**3**), luteolin-3′,4′ -dimethyl ether (**4**), and 5,3′-dihydroxy-6,7,4′,5′-tetramethoxyflavone (**5**).

### SNEEDS Formulation

Three different SNEDDS groups, of seven runs each, containing PEG 400 as a cosurfactant, tween 80 as a surfactant and one of the studied oils (olive oil, linoleic acid, and oleic acid) were prepared and characterized for the globule size and PDI, and the obtained results are represented in Table 1.

The aim was to develop a nano-emulsion with the lowest globule size. The united states pharmacopeia (USP) <729> stated that the mean globule size of an injectable emulsion should be <500 nm (Pharmacopeia., 2019). Injectable emulsions that reach a diameter of 5 μm or more have a high tendency to become entrapped in small capillaries, especially those found in the brain or the lungs, which may cause embolism (Nilsson et al., 2019). In this study, as the concentration of oil was decreased the globule size was decreased, the effect that could be attributed to the availability of more surfactant molecules, at low oil concentration, that adsorbs and form a closely pack surfactant film at the oil/water interface. This effect leads to the formation of a stable system of low interfacial tension and hence small globule size. Based on the obtained results for particle size, SNEDDS formulation which contains 10% linoleic acid, 80% PEG 400, and 10% tween 80 was used to develop a medicated IV formulation containing the isolated compounds.

### Biological Study

The Biological study of the isolated compounds revealed significant vasodilation activity of compounds **1**, **4,** and **5** however, the anti-hypertensive effect was observed only with compounds **1** and **5**.

#### Effects of IV Formulations Contain Compound **1** or **5** on Systolic and Diastolic Blood Pressure and Heart Rate

**FIGURE 2:**
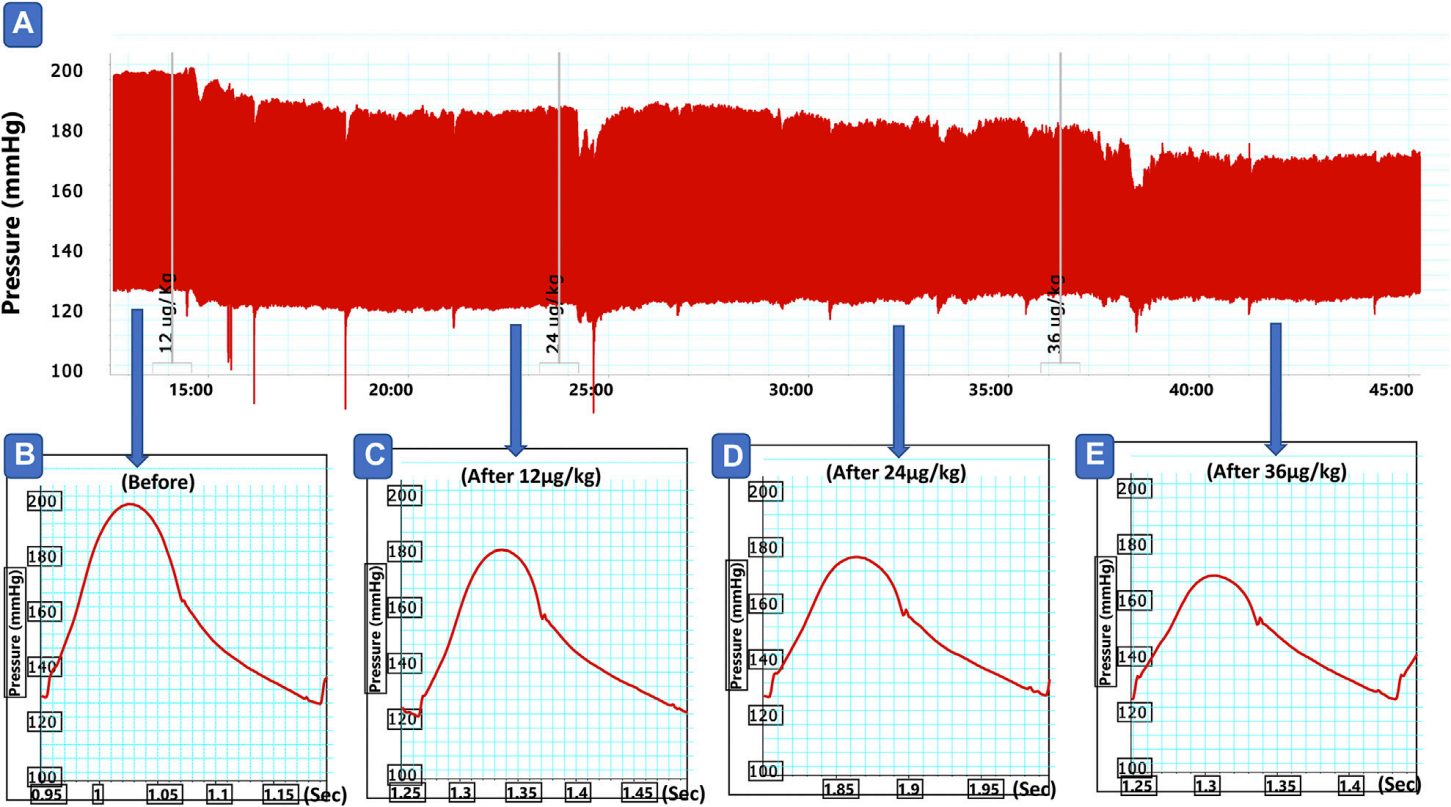
Representative traces of blood hemodynamic invasive recording **(A)**, before **(B)**, and after slow intravenous injection of formulation 1 in doses of 12 **(C)**, 24 **(D)** and **(E)** 36 μg/kg in angiotensin model of hypertensive rats.

**FIGURE 3:**
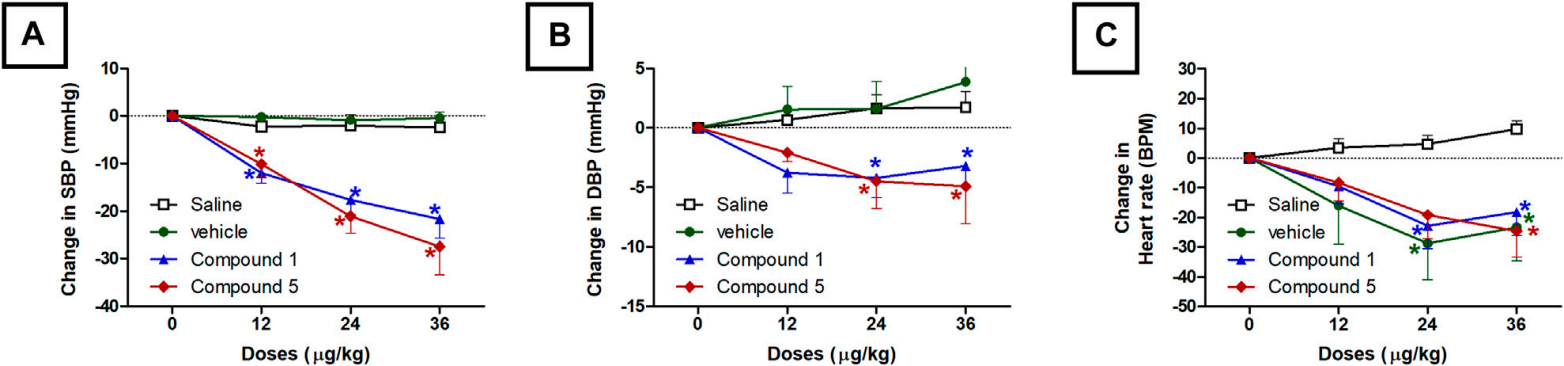
Effect of slow intravenous injection of formulations 1 and 5 on the systolic blood pressure (SBP, **A**), diastolic blood pressure (DBP, **B**), and the heart rate **(C)**. Data presented as mean ± standard error of 6 animals. *p < 0.05, compared with the corresponding vehicle control values; by two Way ANOVA and Bonferroni post hoc test.

As shown in Figures 2,3A, intravenous injection of formulations containing compound **1** or **5** in doses of 12, 24, and 36 μg/kg resulted in a gradual dose-dependent reduction in the elevated systolic blood pressure induced by angiotensin after 10 min of each dose injection (*p* < 0.05). The reduction in diastolic blood pressure was also gradual and reached a plateau with statistical significance at doses 24 and 36 μg/kg (*p* < 0.05, Figure 3B) compared to the group administered the plain IV formulation. Intravenous injection of the plain IV formulation resulted in a significant reduction in heart rate compared to the saline group, while the IV formulations containing compounds **1** or **5** did not produce any further effect on heart rate compared with the plain IV formulation. (Figure 3C). The vasodilating effects of the *P. punctulata* isolated compounds; **1** and **5** were previously reported by our teamwork (Abdallah et al., 2020), but this behavior does not guarantee the antihypertensive efficacy as evidence by the lack of antihypertensive efficacy of compound **4**. Compounds usually have multiple effects and mechanisms, and the net result is a summation of all these effects. The carefully selected vehicle ingredients helped in having a vehicle with a significant reduction of heart rate per se. Among many oils that can be used in SNEDDS preparation, linoleic acid with the reported (Christensen et al., 2005) effect on heart rate was selected. This effect on heart rate rescues the vasodilating effect of compounds isolated from *P. punctulata* from being masked by the reflex tachycardia that commonly associated with the decrease in peripheral resistance by most vasodilators.

#### Effects of IV Formulations Contain Compound **1** or **5** on Pulse Pressure, Dicrotic Notch Pressure, and SDP Difference

**FIGURE 4:**
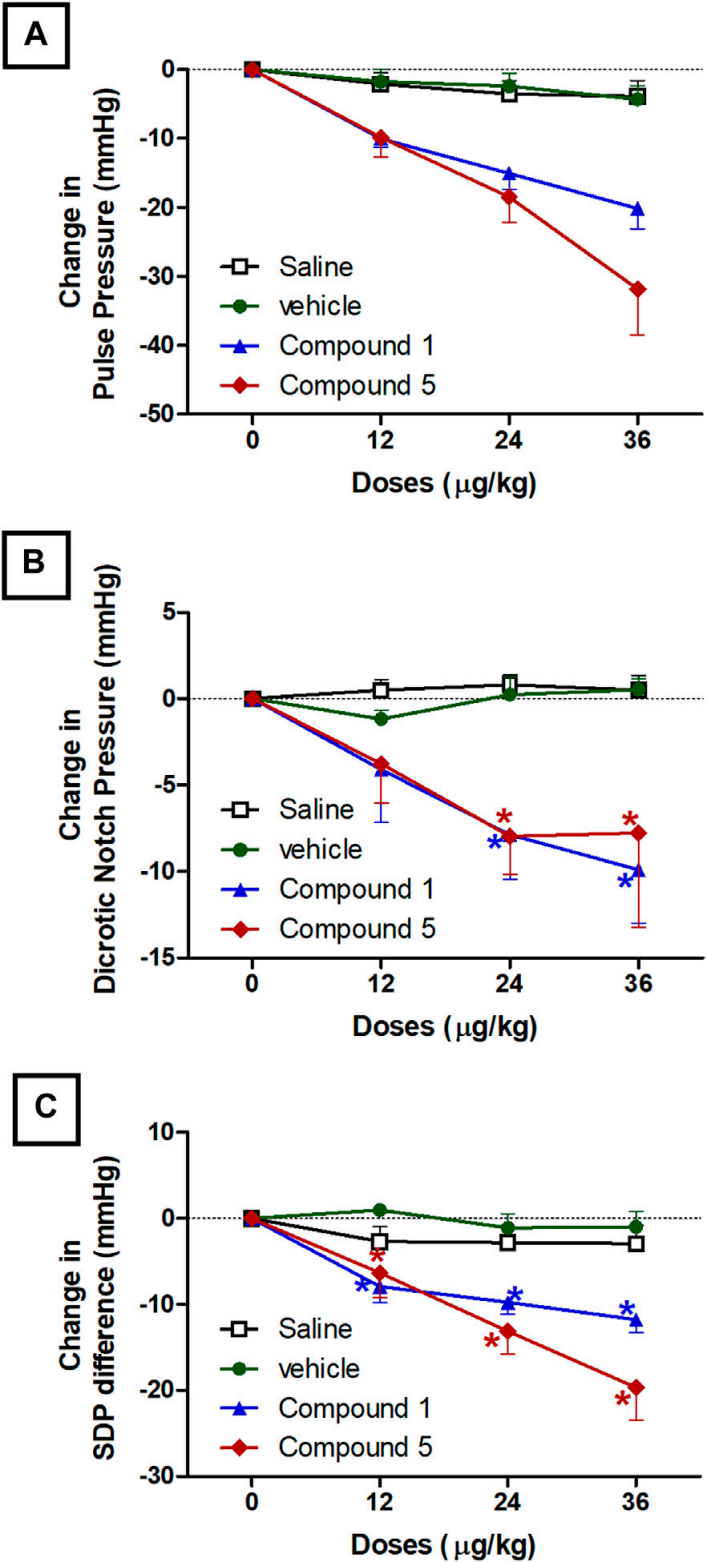
Effect of slow intravenous injection of formulations 1 and 5 on the pulse pressure **(A)**, dicrotic notch pressure **(B)**, and the systolic blood pressure-dicrotic notch pressure difference (SDP-difference, **(C)**. Data presented as mean ± standard error of 6 animals. *p < 0.05, compared with the corresponding vehicle control values; by two Way ANOVA and Bonferroni post hoc test.

Pulse pressure is a marker for increased large arterial stiffness (Safar, 2018) and is a strong predictor of cardiovascular risk and hypertensive organ damage (Litwin et al., 2019). Both formulations contain compounds **1** and **5** resulted in a significant reduction in pulse pressure started after 10 min of intravenous injection of doses 24 and 36 μg/kg compared to plain IV formulation (*p* < 0.05, Figure 4A). The ability of both IV formulations to reduce pulse pressure is related to their ability to improve arterial compliance through reducing systolic blood pressure. Intravenous injection of doses 24 and 36 μg/kg of formulations containing compounds **1** and **5** resulted in a significant reduction in dicrotic notch pressure started after 10 min of injection compared to plain IV formulation (*p* < 0.05, Figure 4B). The dicrotic notch is a ubiquitous feature of the pressure waveform in the aorta. It is universally considered to be a marker for the end of the ventricular ejection period and is used routinely to calculate ejection duration in clinical practice (Nirmalan and Dark, 2014; Gamrah et al., 2020). Moreover, formulations contain compound **1** or **5** resulted in a significant reduction in the difference between systolic and dicrotic pressure (SDP difference) compared to plain IV formulation at doses 12, 24, and 36 μg/kg (*p* < 0.05, Figure 4C). The SDP difference is important in reflecting the coupling between myocardial contractility and a given afterload (Morelli et al., 2020). Previously Senzaki *et al* suggested that increased afterload rather than reduced contractility impairs ventricular-arterial coupling (Senzaki et al., 2006).

#### Effects of IV Formulations Contain Compound **1** or **5** on Ejection Duration, Non-ejection Duration, and Time to Peak

**FIGURE 5:**
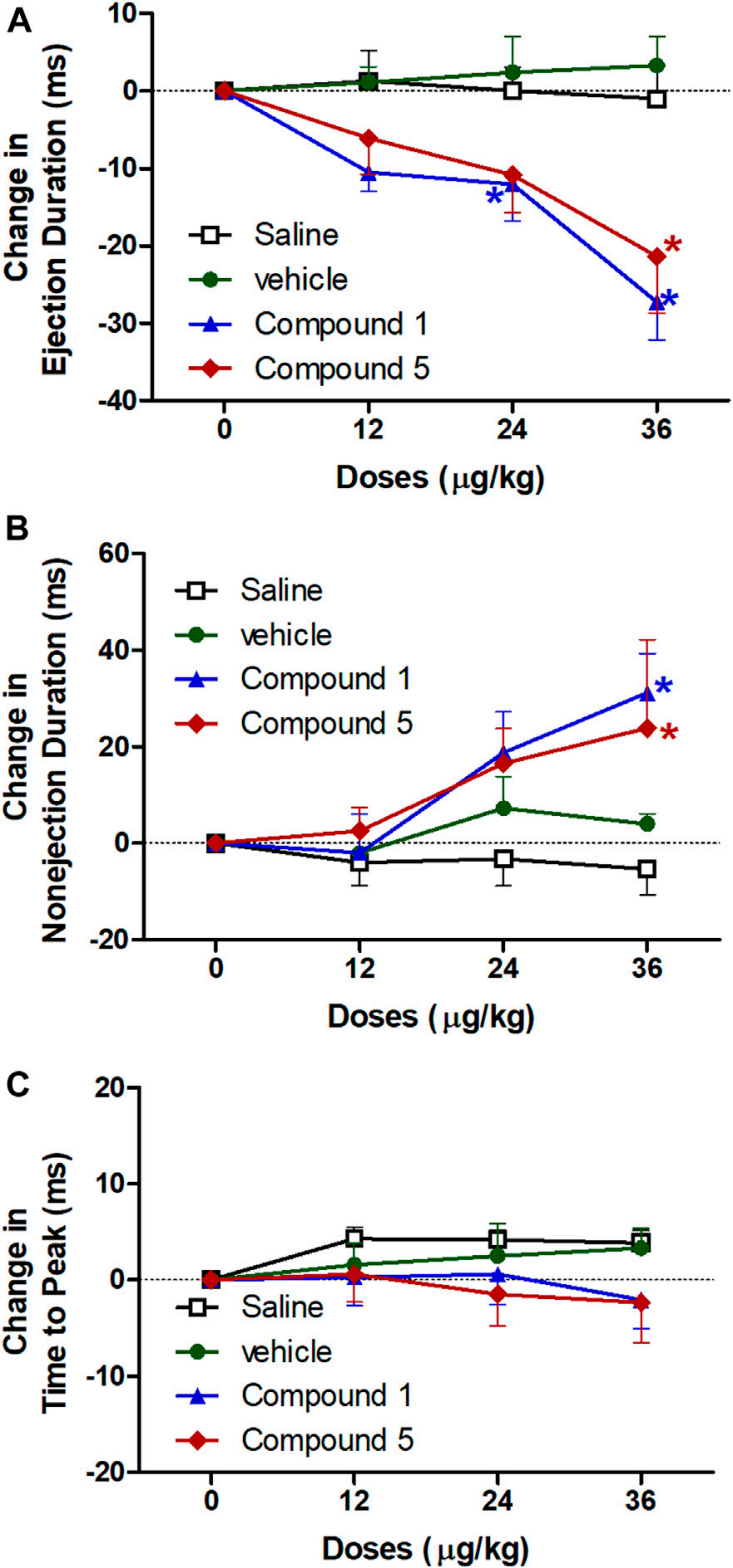
Effect of slow intravenous injection of formulations 1 and 5 on the ejection duration **(A)**, nonejection duration **(B)**, and the time to peak **(C)**. Data presented as mean ± standard error of 6 animals. *p < 0.05, compared with the corresponding vehicle control values; by two Way ANOVA and Bonferroni post hoc test.

Left ventricular systolic ejection duration is the duration of systolic ejection in milliseconds and is a reproducible measure of systolic performance (Avadhani et al., 2018) and its prolongation is a useful sign of aortic stenosis (Kligfield et al., 1984). However, the non-ejection duration reflects the periods when all heart valves are closed. Figure 5 shows that intravenous injection of formulation contains compound **1** resulted in a significant decrease in ejection duration at doses 24 and 36 μg/kg and a significant increase in non-ejection duration at dose 36 μg/kg but no change in time to peak compared to plain IV formulation. While formulation contains compound **5** resulted in a significant decrease in ejection duration and a significant increase in non-ejection duration at dose 36 μg/kg and didn’t change in time to peak compared to plain IV formulation (*p* < 0.05). The ejection duration from the left ventricle has been significantly prolonged in the patients with central systolic blood pressure higher than brachial systolic blood pressure (Bulas et al., 2017) and its prolongation concurrently widens the pulse pressure, which leads to ventricular hypertrophy (Toprak et al., 2009).

#### Effects of IV Formulations Contain Compound **1** or **5** on Cardiac Electrophysiology

**FIGURE 6:**
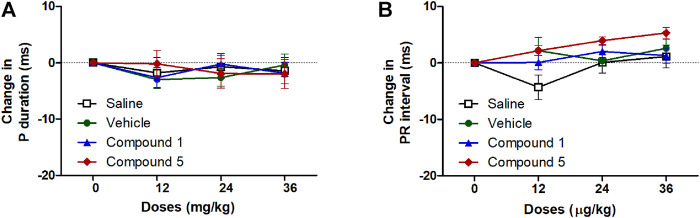
Effect of slow intravenous injection of formulations 1 and 5 on the *p* wave duration **(A)** and PR interval **(B)**. Data presented as mean ± standard error of 6 animals. *p < 0.05, compared with the corresponding vehicle control values; by two Way ANOVA and Bonferroni post hoc test.

**FIGURE 7:**
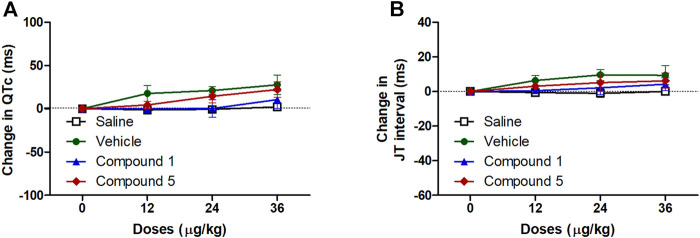
Effect of slow intravenous injection of formulations 1 and 5 on the QTc interval **(A)** and the JT interval **(B)**. Data presented as mean ± standard error of 6 animals. *p < 0.05, compared with the corresponding vehicle control values; by two Way ANOVA and Bonferroni post hoc test.

ECG signals and the information obtained through the analysis of these signals constitute the main source of diagnosis for many cardiovascular system diseases including hypertension (Yanık et al., 2020). In the current study injection of formulations containing compound **1** or **5** did not significantly affect atrial conductivity or the propagation of the impulse through the AV node and the conduction system to the ventricles as they did not affect the P-wave duration or PR interval compared to plain IV formulation (Figure 6). Similarly, the entire ventricular activity from the beginning of ventricular depolarization through the plateau phase to the ventricular repolarization was not affected by intravenous injection of formulations containing compound **1** or **5** as it had no significant effect on either QTc or JT intervals compared to plain IV formulation in hypertensive rats infused with angiotensin II (Figure 7).

**FIGURE 8:**
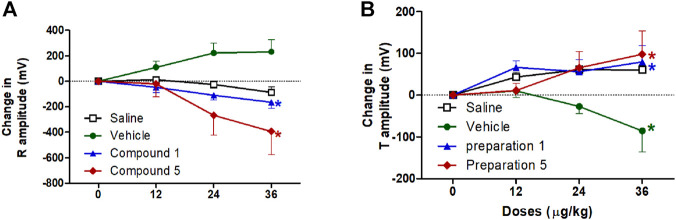
Effect of slow intravenous injection of formulations 1 and 5 on the R amplitude **(A)** and the T amplitude **(B)**. Data presented as mean ± standard error of 6 animals. *p < 0.05, compared with the corresponding vehicle control values; by two Way ANOVA and Bonferroni post hoc test.

On the other hand, vehicle intravenous injection resulted in a reduction in T wave amplitude, this reduction is significantly different from the saline group at dose 36 μg/kg (*p* < 0.05, Figure 8B). Both formulations contain compound **1** or **5** resulted in a significant decrease in R wave amplitude which represents early ventricular depolarization and an increase in T wave amplitude which reflects ventricular repolarization compared to vehicle group only at dose 36 μg/kg of formulation 5 (*p* < 0.05, Figure 8). Its obvious from these results that the effect of formulations containing compound 1 or 5 is on the amplitudes not the duration of the electrical vectors reflecting ventricular activity.

#### Effects of IV Formulations Contain Compound **1** or **5** on Serum Levels of Sodium and Potassium

**FIGURE 9:**
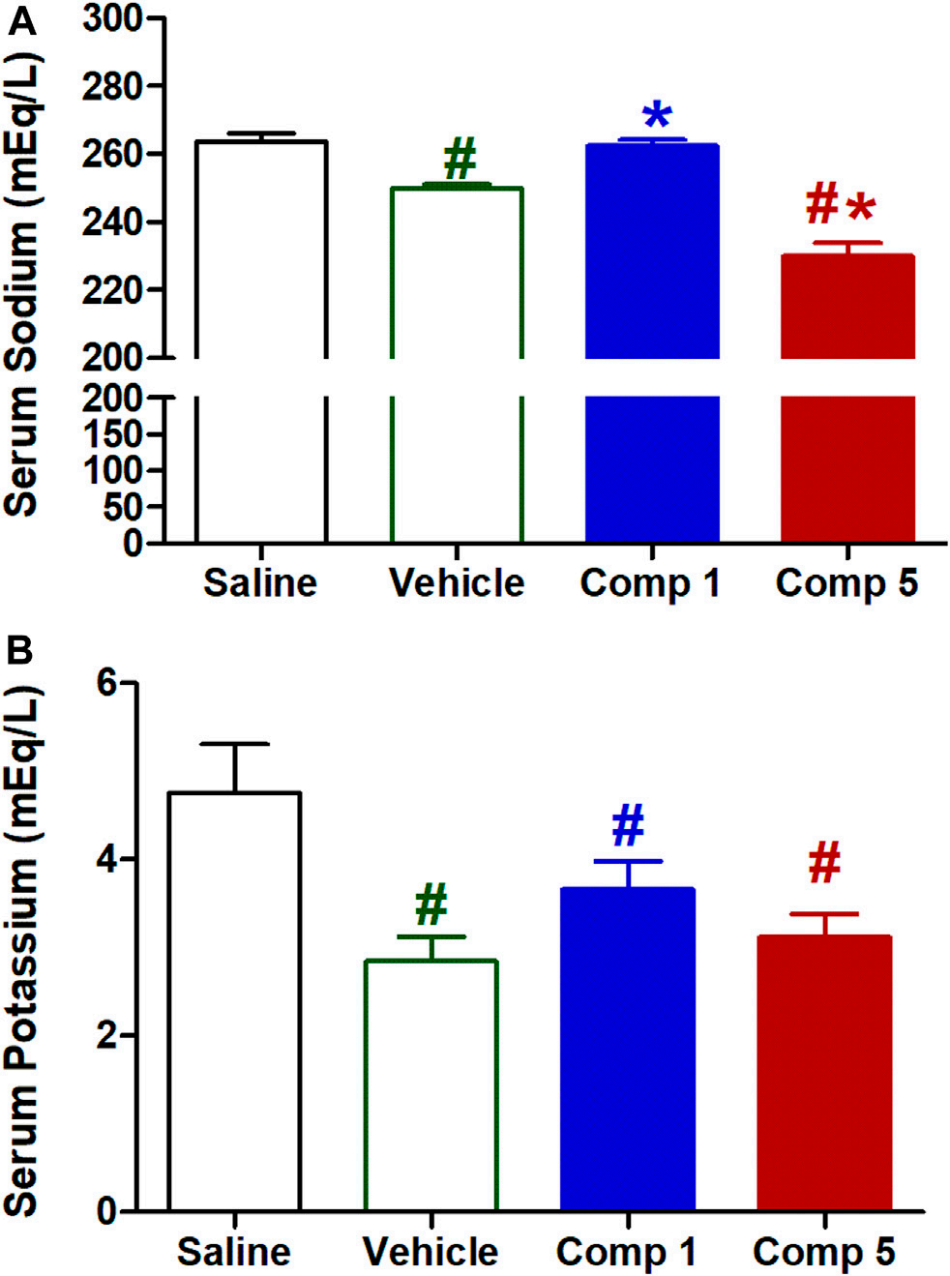
Effect of slow intravenous injection of formulations 1 and 5 on serum sodium **(A)** and potassium **(B)** levels. Data presented as mean ± standard error of 6 animals. *p < 0.05, compared with the corresponding vehicle control values, #*p* < 0.05, compared with the corresponding saline control values; by One Way ANOVA and Newman Keuls post hoc test.

Sodium and potassium are two important electrolytes that are closely associated with changes in cardiovascular structures and functions (Zhao et al., 2018). High serum sodium can be a marker of risk for increased pulse pressure, a surrogate index of arterial stiffness, in individuals at high risk for cardiovascular events (Nowak et al., 2019). As shown in Figure 9A, intravenous injection of the preparations vehicle resulted in a significant reduction in serum sodium level (*p* < 0.05) compared to the saline group. While compound **1** formulation significantly increased serum sodium compared with vehicle (*p* < 0.05) returning it to the initial level of saline control, compound **5** preparation produced a significant further reduction in serum sodium compared with the vehicle (*p* < 0.05).

On the other hand, intravenous injection of the preparations vehicle resulted in a significant reduction in serum potassium level (*p* < 0.05, Figure 9B) compared to the saline group. While neither compound **1** nor **5** preparations produced significant further reductions in serum potassium levels, they still significantly reduced from the saline control (*p* < 0.05). The ability of formulation contains compound **5** to reduce serum sodium level could arise from its direct vasodilation which increases renal blood flow and then increasing glomerular filtration. However, further studies are required to determine the underlying mechanisms involved with sodium and potassium excretion and the precise location of action in the nephron.

In conclusion, vasodilators produced reflex tachycardia and an increase in heart rate that masks its anti-hypertensive effect. The formulation contains compounds **1** or **5** in IV SNEDDS containing 10% linoleic acid caused significant antihypertensive effect through vasodilatation and decreasing peripheral resistance without reflex tachycardia. This could be detected through the ability of this formula to reduce the elevated diastolic, pulse pressure, dicrotic notch pressure, and the systolic–dicrotic notch pressure difference and through modulation of ventricular electrical activity. The studied nano-pharmaceutical formulation suggesting them as effective medications in hypertensive emergencies, after clinical evaluation.

## Data Availability

The original contributions presented in the study are included in the article/Supplementary Material, further inquiries can be directed to the corresponding author.
